# Role of Cattle Movements in Bovine Tuberculosis Spread in France between 2005 and 2014

**DOI:** 10.1371/journal.pone.0152578

**Published:** 2016-03-28

**Authors:** Aurore Palisson, Aurélie Courcoul, Benoit Durand

**Affiliations:** 1 University Paris Sud, Orsay, France; 2 University Paris Est, Anses, Laboratory for Animal Health, Epidemiology Unit, Maisons-Alfort, France; INIAV, I.P.- National Institute of Agriculture and Veterinary Research, PORTUGAL

## Abstract

Live animal movements are a major transmission route for the spread of infectious agents such as *Mycobacterium bovis*, the main agent of bovine Tuberculosis (bTB). France became officially bTB-free in 2001, but *M*. *bovis* is still circulating in the cattle population, with about a hundred of outbreaks per year, most located in a few geographic areas. The aim of this study was to analyse the role of cattle movements in bTB spread in France between 2005 and 2014, using social network analysis and logistic regression models. At a global scale, the trade network was studied to assess the association between several centrality measures and bTB infection though a case-control analysis. The bTB infection status was associated with a higher in-degree (odds-ratio [OR] = 2.4 [1.1–5.4]) and with a higher ingoing contact chain (OR = 2.2 [1.0–4.7]). At a more local scale, a second case-control analysis was conducted to estimate the relative importance of cattle movements and spatial neighbourhood. Only direct purchase from infected herds was shown to be associated with bTB infection (OR = 2.9 [1.7–5.2]), spatial proximity to infected herds being the predominant risk factor, with decreasing ORs when distance increases. Indeed, the population attributable fraction was 12% [5%–18%] for cattle movements and 73% [68%–78%] for spatial neighbourhood. Based on these results, networks of potential effective contacts between herds were built and analysed for the three major spoligotypes reported in France. In these networks, the links representing cattle movements were associated with higher edge betweenness than those representing the spatial proximity between infected herds. They were often links connecting distinct communities and sometimes distinct geographical areas. Therefore, although their role was quantitatively lower than the one of spatial neighbourhood, cattle movements appear to have been essential in the French bTB dynamics between 2005 and 2014.

## 1. Introduction

Bovine Tuberculosis (bTB) is a chronic disease mainly due to *Mycobacterium bovis*. It can affect many hosts, in particular humans and cattle [1]. France has been recognized Officially Tuberculosis Free since 2001 (UE Decisions n° 2001/26/EC). However, this status does not imply that the disease has been eradicated: 112 outbreaks were reported in France in 2013, most of them clustered in a few geographical areas [2]. The goal of the national action plan for bTB is to eradicate the infection but current surveillance and control measures have so far not succeeded in achieving this objective. In order to better target control measures, more knowledge on the major determinants of bTB spread in France is needed.

The transmission routes of bTB between farms are multiple. In Great Britain, cattle movements have been shown to be one of the main between-herd transmission routes [3–5], allowing bTB to spread over both short- and long-range distances and to be introduced into new geographical areas. Carrique-Mas *et al*. [6] also concluded that cattle movements were the only bTB source for restocked herds with no bTB history. In France, about 5 million cattle are exchanged every year between around 250,000 farms [7,8]. This large number of animals sent from a herd to another one could be a major determinant of between-herd bTB spread. However, local transmission routes also exist: direct contacts between cattle of neighbouring herds despite the fences [9], infection through a common infected wildlife source [10] or residual infection persisting within the herd after a first infection [11]. Depending on whether the predominant between-herd transmission routes are through cattle movements or through local transmission processes, the most appropriate surveillance and control measures will be different.

Social network analysis methods are often used to study cattle movements and disease spread [7–9,11–14]. In the network, herds are represented by nodes, and animal movements between herds are represented by directed (sometimes weighted) links between the nodes. Dutta *et al*. [7] and Rautureau *et al*. [8] previously described the French cattle movement network. Both showed the presence of a Giant Strong Component (GSC) in the network, i.e. a subnetwork in which all nodes are mutually accessible by following the direction of the links [15]. About 40% of all nodes of the network are included in the GSC for yearly aggregated networks. If a disease is introduced into any node of this subnetwork, it could potentially reach all the other nodes [8]. Indeed, Kao *et al*. [16] and Kiss *et al*. [17] showed that GSC size was a good predictor to estimate a potential epidemic size. Thus, cattle movements could be a major route of bTB transmission in France, especially because of the difficulty in detecting the infection (clinical signs are rare and non-specific and diagnostic tests are imperfect). That is why mandatory pre-movement tests exist in several countries, including European countries and the United States.

The most commonly used indicators for network analysis are the centrality measures. The degree is an indicator of how connected a node (i.e. a herd) is, within a graph. If cattle movements have a strong role in bTB spread, highly connected nodes should be more likely to get the infection: differences in centrality measures between bTB-infected and non-infected herds may thus be expected. Therefore, in the first part of our study, we assessed the association between some network centrality indicators and the bTB status of nodes (i.e. herds) in order to determine whether cattle movements have a strong role in bTB spread.

In France, as the prevalence of bTB is low, the bTB status of a given herd is expected to be related to the bTB status of the herds it has been in contact with, i.e. its neighbourhood. This herd neighbourhood consists of its neighbourhood in the cattle trade network (termed below “network neighbourhood”) but also of its geographical neighbourhood (termed below “spatial neighbourhood”): in France, most infected herds are clustered in a few geographical areas and local transmission is assumed to play a role in bTB spread. The bTB status of a herd is then expected to be associated with the bTB statuses of the herds located in the surrounding areas. To quantify the relative importance of cattle trade and local transmission in bTB spread, we therefore assessed, in the second part of our study, the association between the bTB status of herds and their exposure to bTB-infected herds in their network and spatial neighbourhood, respectively.

However, even if cattle trade had a quantitatively smaller role in bTB spread compared to local transmission, it should be kept in mind that cattle trade has the potential to allow bTB to spread over long-range distances. In the third and last section of our work, a network of potential effective contacts (contacts which may have led to the transmission of the infection) between bTB-infected herds was generated for each of the three major French molecular types. These three networks were analysed to assess the respective roles of network and spatial neighbourhood in bTB overall spread in France between 2005 and 2014 through a comparison of the respective roles, in the network, of the links representing network neighbourhood, and of the links representing spatial neighbourhood to connect bTB-infected herds.

## 2. Materials and Methods

### 2.1. Ethical statement

bTB is a notifiable disease under mandatory surveillance and control in France. Official methods for detection are skin tests (the single intradermal tuberculin test–SITT, and the single intradermal comparative cervical tuberculin test–SICCT) and meat inspection. Official methods for confirmatory diagnosis are bacteriology and histology. All the datasets included in this study are issued from animals analysed within an official context. No purpose killing of animals was performed for this study. All datasets were in complete agreement with French and European regulations. No ethical approval was necessary.

### 2.2. Data

In France, cattle are individually identified, and farmers report each animal birth, movement and death within the seven days following the event. These reports are registered in the French cattle tracing system database named “Base de Données Nationale d'Identification” (BDNI). A subset of this database was provided by the French Ministry of Agriculture for the 2005–2014 (inclusive) period, corresponding to nine production campaigns (i.e. periods starting on the 1^st^ of July and ending on the 30^th^ of June of the following year). This dataset included (i) individual cattle movements (animal ID number, herd ID number, date and cause of movement: birth, sale or slaughter), (ii) individual data (date of birth, sex, breed and date of the first calving), and (iii) herd geolocation at the commune level (the smallest administrative French subdivision). For a given time period, this dataset allowed constructing a network of cattle movements, with herds as nodes and movements of animals between distinct herds as directed links (all of the movements between a given pair of herds being aggregated into a single link).

For each production campaign, herds were classified into 4 herd types, based on three production indicators, the average annual number of animals per racial type (beef, dairy, mixed), the annual number of calvings par racial type and the annual number of males sold. The four herd types were: dairy herd, beef herd, mixed herd and other herd (see **S1 File** for the full definitions).

Concerning bTB data, herd ID numbers and dates of disease notification were obtained from the French Ministry of Agriculture, for all the herds reported as bTB-infected during the nine production campaigns (i.e. July 2005-June 2014). Strains typing results (spoligotype) were obtained from the French National Reference Laboratory.

In order to assess the association between indicators and bTB infection, two case-control studies were performed. Cases were bTB infected herds for which the spoligotype was available and was observed in >1 case (thus excluding cases that could not be linked to any other case, based on typing results). In case of multiple bTB breakdowns in the same herd during the study period, only the first report was considered. For each case herd, one control herd was randomly chosen among bTB non-infected herds of the same department, verifying that herd size was >0 at the date of bTB notification in the matched case. For each herd (cases and controls), the herd size, the herd type and the Euclidean distance to the closest herd reported as infected with the same spoligotype (each herd being geolocated at the centroid of its commune) were calculated as adjustment variables.

### 2.3. Network centrality indicators and bTB infection

The association between network centrality indicators and bTB infection status was analysed using a multivariate logistic regression model. The outcome variable was the bTB infection status (case or control). Predictor variables were static and dynamic centrality measures: the in-degree (number of incoming links, i.e. the number of herds that sent cattle to the node), the out-degree (number of outgoing links, i.e. the number of herds to whom the node sent cattle), the node betweenness centrality (number of shortest paths from all nodes to all others going through this node) and the ingoing contact chain (number of herds in direct or indirect contact though successive movements ending in the herd, and taking into account the temporal sequence of these movements) [15,18,19]. Three adjustment variables were taken into account as predictor variables: herd size, herd type and Euclidean distance to the closest herd reported as infected with the same spoligotype. The matching variable (the department) was also included in the logistic model.

In France, bTB-infected herds are subjected to movement restrictions as soon as infection is detected. This would obviously bias the analysis if the whole dataset of cases and controls was used to fit the model. To avoid this bias, the above centrality indicators were computed using the network built from movements having occurred between the 1^st^ of July 2005 and the 30^th^ of June 2010. The model was then fit using cases and their associated controls for which the infection had been detected between the 1^st^ of July 2010 and the 30^th^ of June 2014.

Predictor variables included in the multivariate regression model were selected based on univariate regression models, when the *p*-value was <0.20. Spearman correlation coefficient was calculated between pairs of variables thus selected. Pairs of variables for which the value was >0.70 were not simultaneously included in the same multivariate model, the two competing models being instead compared based on the Akaike Information Criterion (AIC, a difference >4 being considered indicative of the superiority of one model). The quantitative variables were cut in 4 classes based on the quartiles of their distributions. For each multivariate model, the variance inflation factor (VIF) was checked to be lower than 5, indicating the absence of significant multi-collinearity between predictor variables. The predictive quality of the model was assessed by computing the area under the ROC curve.

The values of network centrality indicators are, by definition, non-independent (especially for nodes that are close in the network). For example, the in-degree of a node is directly related to the out-degree of its neighbours. Because of this non-independence of observations for network centrality indicators, a bootstrap procedure [20] was implemented to assess the significance of the corresponding model coefficients. One thousand networks were generated by random permutation of the original adjacency matrix. The multivariate logistic regression model was fitted for each of these networks, resulting in 1,000 estimates of model parameters. For each network centrality indicator, the empirical *p*-value was then the proportion of these 1,000 estimates for which the value was higher (or lower) than the ones obtained using the real network.

### 2.4. Network and spatial neighbourhood and bTB infection

The association between bTB infection and exposure variables based on herd neighbourhood was analysed using a multivariate logistic model. The outcome variable was the bTB infection status (case or control) and two types of exposure variables were included as predictor variables: (i) variables based on the herd neighbourhood in the cattle trade network (termed below “network neighbourhood”), and (ii) variables based on herd location relative to bTB infected herds (termed below “spatial neighbourhood”). The matching variable (the department) was also included in the logistic model.

A herd Y was considered exposed to bTB infection in its network neighbourhood of order n if there existed at least one bTB-infected herd X, and n non bTB-infected herds x_1_…x_n_, such that X sold animals to x_1_, which later sold animals to x_2_, and so on until x_n_ from which animals were eventually sold to Y. A further constraint was taken into account if Y was bTB-infected: the spoligotypes found in X and Y had to be identical. Moreover the time interval between the first and the last animal movement (X to x_1_ and x_n_ to Y) had to be less than 5 years. Three binary exposure variables were thus defined corresponding to network neighbourhood of order 0 (direct neighbourhood), 1 and 2.

A herd Y was considered exposed to bTB infection in its spatial neighbourhood of order k if there existed at least one bTB-infected herd in the k^th^-order spatial neighbourhood of its commune (the smallest administrative French subdivision). The 0^th^-order spatial neighbourhood of a commune was the commune itself; its 1^st^-order spatial neighbourhood corresponded to its adjacent communes; its 2^nd^-order spatial neighbourhood was the adjacent communes of its 1^st^-order spatial neighbourhood (considering communes which were not already included in the 0^th^- and 1^st^-order neighbourhoods), etc. Five binary variables were thus defined corresponding to spatial neighbourhood of order 0 to 4. Again, a further constraint was taken into account if Y was bTB-infected: the spoligotypes found in X and Y had to be identical. Moreover the time interval (absolute value) between bTB detection in X and Y had to be less than 5 years.

Two adjustment variables were included as predictor variables: the herd size (cut into 4 classes according to the quartiles of the distribution) and the herd type. The variance inflation factor (VIF) was checked to be lower than 5 to verify the absence of significant multicollinearity between the predictor variables. The predictive quality of the model was assessed by computing the area under the ROC curve. Finally, the relative weights of network and spatial neighbourhoods in bTB infection risk were quantified using the corresponding population attributable fractions.

### 2.5. Networks of potential bTB effective contacts

For each of the major spoligotypes identified in France (>50 infected herds between 2005 and 2014), a network of potential effective contacts was generated and analysed to assess the respective roles of network and spatial neighbourhood in bTB overall spread. Nodes were the bTB-infected herds, and links corresponded to the above network and spatial neighbourhood relationships, selecting neighbourhood orders for which a significant association with bTB status has been evidenced in the previous steps. Links representing network neighbourhood were directed, as cattle movements were oriented. Links representing spatial neighbourhood were bidirectional. Two attributes were associated to each node: the year of the bTB report and the department. One attribute was associated to each link: the link type (network or spatial neighbourhood).

Several indicators were then calculated: (i) the number of nodes, (ii) the number of links of each type (network or spatial neighbourhood), (iii) the number of components and, for each component of more than 10 nodes, (iv) the number of communities obtained using the edge-betweenness communities detection algorithm [21]. Wilcoxon’s test was used to analyse the association between edge betweenness (number of shortest paths from all nodes to all others going through this link) and link type (network or spatial neighbourhood). Similarly, for each component of ≥10 nodes, Fisher’s test was used to assess the association between the link type and the fact that the link connected two nodes of the same community or two nodes of distinct communities.

All the analyses were performed with the packages igraph [22], EpiContactTrace [23], car [24], pROC [25] and AF [26] for R 3.1 [27].

## 3. Results

### 3.1. Network centrality indicators and bTB infection

A total number of 262,453 herds sold at least an animal between July 2005 and June 2010, representing 262,453 nodes and 7,328,862 links on the network. There were 789 herds reported as bTB-infected in France between June 2005 and July 2014. Among these, the spoligotype was available and observed in at least two herds in 648 infected herds. Approximately half of these 648 bTB-infected herds (N = 296) were detected between 2010 and 2014 (July 2010-June 2011: 54 herds reported as infected; July 2011-June 2012: 79; July 2012-June 2013: 73; July 2013-June 2014: 90). Hence, the association between network centrality indicators and bTB infection status was analysed using these 296 cases and 296 associated controls.

Variable selection led to include 3 network centrality indicators in the multivariate model: the in-degree, the node betweenness, and the ingoing contact chain (p<0.20 in univariate models). An expected strong correlation (Spearman coefficient of 0.76) was observed between the in-degree and the ingoing contact chain. Two multivariate models were thus compared: the first one with the in-degree and the node betweenness (**Table 1**), and the second one with the ingoing contact chain and the node betweenness (**Table 2**). Both models were adjusted on the Euclidian distance to the nearest bTB-infected herd with the same spoligotype, the herd size and the herd type. For both models, the maximal VIF was <5 (1.3 in both cases). The AICs of the two models were close, with an AIC of 681 for the first model (which included the in-degree) and an AIC of 684 for the second model (which included the ingoing contact chain).

**Table 1:** Multivariate logistic regression model of bTB infection status according to network centrality indicators: model including the in-degree (complete model, AIC = 681).

		Number of cases	Number of controls	Odds-ratio (95% CI)	*p*-value
In-degree					
	[0–1]	58	89	Ref	
	]1–3]	49	64	1.0 (0.5–2.0)	0.57
	]3–9]	87	74	1.7 (0.8–3.5)	0.10
	**]9–7 280]**	**102**	**69**	**2.4 (1.1–5.4)**	**0.01**
Node betweenness					
	0	44	64	Ref	
	]0–1.28x10^-6^]	56	77	0.9 (0.4–2.1)	0.56
	]1.28x10^-6^–1.03x10^-5^]	78	63	1.2 (0.5–2.8)	0.30
	]1.03x10^-5^–0.0733]	118	92	1.4 (0.6–3.1)	0.22
Distance to the nearest infected herd (km)					
	0	133	21	Ref	
	**]0–8.46]**	**89**	**57**	**0.2 (0.1–0.4)**	**< 0.001**
	**]8.46–24.7]**	**34**	**112**	**0.03 (0.01–0.05)**	**< 0.001**
	**]24.7–710]**	**40**	**106**	**0.01 (0.01–0.03)**	**< 0.001**
Herd size					
	[0–18.1]	58	84	Ref	
	**]18.1–41.8]**	**77**	**64**	**2.5** (1.1–6.3)	**0.04**
	]41.8–74.4]	75	68	2.3 (0.9–6.0)	0.08
	]74.4–374]	86	80	1.8 (0.7–4.9)	0.25
Herd type					
	Beef	181	140	Ref	
	Other	66	77	0.9 (0.4–2.2)	0.79
	**Dairy**	**33**	**67**	**0.4 (0.2–0.7)**	**0.003**
	Mixed	16	12	1.1 (0.4–3.1)	0.89

**Table 2:** Multivariate logistic regression model of bTB infection status according to network centrality indicators: model including the ingoing infection chain (complete model, AIC = 684).

		Number of cases	Number of controls	Odds-ratio (95% CI)	*p*-value
Ingoing Contact Chain					
	[0–11,500]	58	90	Ref	
	]11,500–192,000]	76	72	1.7 (0.9–3.5)	0.12
	]192,000–206,000]	76	72	1.4 (0.7–3.0)	0.34
	**]206,000–220,000]**	**86**	**62**	**2.2 (1.0–4.7)**	**0.04**
Node betweenness					
	0	44	64	Ref	
	]0–1.28x10^-6^]	56	77	0.8 (0.4–1.8)	0.72
	]1.28x10^-6^–1.03x10^-5^]	78	63	1.2 (0.5–2.6)	0.33
	]1.03x10^-5^–0.0733]	118	92	1.5 (0.7–2.8)	0.12
Distance to the nearest infected herd (km)					
	0	133	21	Ref	
	**]0–8.46]**	**89**	**57**	**0.2 (0.1–0.4)**	**< 0.001**
	**]8.46–24.7]**	**34**	**112**	**0.03 (0.01–0.05)**	**< 0.001**
	**]24.7–710]**	**40**	**106**	**0.01 (0.01–0.03)**	**< 0.001**
Herd size					
	[0–18.1]	58	84	Ref	
	**]18.1–41.8]**	**77**	**64**	**2.9 (1.2–7.3)**	**0.02**
	**]41.8–74.4]**	**75**	**68**	**2.7 (1.1–7.0)**	**0.04**
	]74.4–374]	86	80	2.2 (0.8–6.0)	0.12
Herd type					
	Beef	181	140	Ref	
	Other	66	77	1.1 (0.4–2.6)	0.91
	**Dairy**	**33**	**67**	**0.4 (0.2–0.8)**	**0.007**
	Mixed	16	12	1.2 (0.4–3.5)	0.70

An association between the highest class of in-degree (i.e. the upper quartile) and bTB infection was observed with an OR of 2.4 [1.1–5.4]. Similarly, the upper quartile of the ingoing infection chain was significantly associated with bTB status (OR: 2.2 [1.0–4.7]) (see **Table 1**, **Table 2** and **S1 Fig** and **S2 Fig**). In both models, each of the three adjustment variables was significantly associated with bTB status. An increasing distance to the closest herd reported as infected was associated with a decreasing infection risk. Medium herd size classes were associated with an increased bTB risk. Dairy herds were significantly less often infected than beef herds. This last result was expected since dairy cows are, on average, culled earlier than beef cows in France. Furthermore, dairy cattle have less often access to pasture than beef cattle. Dairy herds have thus less risk to be infected than beef herds in France. Finally, comparable results were obtained when using the infected herds reported between July 2010 and June 2011, instead of the July 2010-June 2014 period.

The area under the ROC curve was 0.86 [0.83–0.89] for the model including the in-degree (**S3 Fig**), and 0.85 [0.82–0.88] for the model including the ingoing contact chain, indicating a “good” fit quality for both models (**S4 Fig**).

### 3.2. Network and spatial neighbourhood and bTB infection

The association between bTB infection and network or spatial neighbourhood was analysed using the 648 bTB-infected herds reported as infected between 2005 and 2014 for which the spoligotype was available and observed in >1 herd, and their 648 associated controls.

The bTB infection status was significantly associated with the network neighbourhood of order 0 (direct neighbourhood, OR = 2.9 [1.7–5.2]), as well as with the spatial neighbourhood of order 0 to 3 with decreasing ORs, from 4.2 to 1.7 (**Table 3**). Again, the two adjustment variables included in the model were significantly linked to bTB infection status: increasing ORs were observed with increasing herd sizes, and dairy herds were significantly less often infected than beef herds.

**Table 3:** Multivariate logistic regression model of bTB infection status according to the exposure to bTB-infected herds in the network and spatial neighbourhood.

		Number of cases	Number of controls	Odds-ratio (95% CI)	*p*-value
Exposure to bTB-infected herds in the network neighbourhood					
	**Order 0**	**116**	**31**	**2.9 (1.7–5.2)**	**< 0.001**
	Order 1	172	78	1.3 (0.8–2.0)	0.30
	Order 2	249	161	1.2 (0.8–1.7)	0.44
Exposure to bTB-infected herds in the spatial neighbourhood					
	**Order 0**	**266**	**42**	**4.2 (2.8–6.5)**	**< 0.001**
	**Order 1**	**411**	**114**	**3.7 (2.4–5.7)**	**< 0.001**
	**Order 2**	**441**	**166**	**3.1 (2.0–4.9)**	**< 0.001**
	**Order 3**	**455**	**233**	**1.7 (1.1–2.6)**	**0.03**
	Order 4	435	270	0.8 (0.5–1.3)	0.38
Herd size					
	[0–18.1]	128	196	Ref	
	**]18.1–41.8]**	**165**	**159**	**2.7 (1.5–4.8)**	**0.001**
	**]41.8–74.4]**	**170**	**154**	**3.1 (1.7–5.9)**	**< 0.001**
	**]74.4–374]**	**185**	**139**	**4.3 (2.2–8.2)**	**< 0.001**
Herd type					
	Beef	391	304	Ref	
	Other	155	186	1.5 (0.9–2.6)	0.14
	**Dairy**	**67**	**140**	**0.4 (0.2–0.6)**	**< 0.001**
	Mixed	35	18	1.3 (0.6–2.8)	0.51

The area under the ROC curve (computed for the complete model described in **Table 3**) was 0.86 [0.84–0.88], indicating a “good” fit quality (**S5 Fig**). No spatial aggregation of the deviance residuals was observed in the corresponding map (**S6 Fig**).

The complete multivariate model was finally used to compute the population attributable fractions associated with the network neighbourhood and with spatial neighbourhood. Results showed a predominance of spatial neighbourhood on bTB infection risk, with an estimated population attributable fraction of 12% [5%–18%] for network neighbourhood (order 0), and of 73% [68%–78%] for the spatial neighbourhood (order 0 to 3).

### 3.3. Networks of potential bTB effective contacts

The respective roles of network and spatial neighbourhood in bTB overall spread were analysed for the 3 spoligotypes that were identified in more than 50 herds reported as infected between 2005 and 2014: SB0120 (296 herds), SB021 (71 herds) and SB0134 (67 herds). For each of these spoligotypes, a network of potential effective contacts was built using the neighbourhood exposure variables found to be significantly associated with the bTB infection status in the previous step: network neighbourhood of order 0, and spatial neighbourhood of order 0 to 3.

The SB0120 network was composed of 32 components, two of which contained more than 10 nodes: a smaller component of 99 nodes (33% of all the nodes of the SB0120 network), and a larger component of 157 nodes (53% of the nodes of the SB0120 network). The SB021 network was made of 6 components, one of which included most of the nodes (66 of 71 nodes). The SB0134 network contained 10 components, two of which contained >10 nodes (**Table 4**). As expected, in each of the three networks, most of the links were spatial neighbourhood links.

**Table 4:** Description of the networks of the 3 spoligotypes which were identified in more than 50 infected herds in France, 2005–2014.

		Number of nodes	Number of links: network neighbourhood	Number of links: spatial neighbourhood	Number of communities of more than 10 nodes
SB0120		296	39	4360	-
	Component 1	99 (33%)	13	1919	2
	Component 2	157 (53%)	25	2428	3
SB021		71	9	680	-
	Component 1	66 (93%)	9	680	3
SB0134		67	5	367	-
	Component 1	44 (66%)	4	326	2
	Component 2	12 (18%)	0	39	1

**Fig 1:**
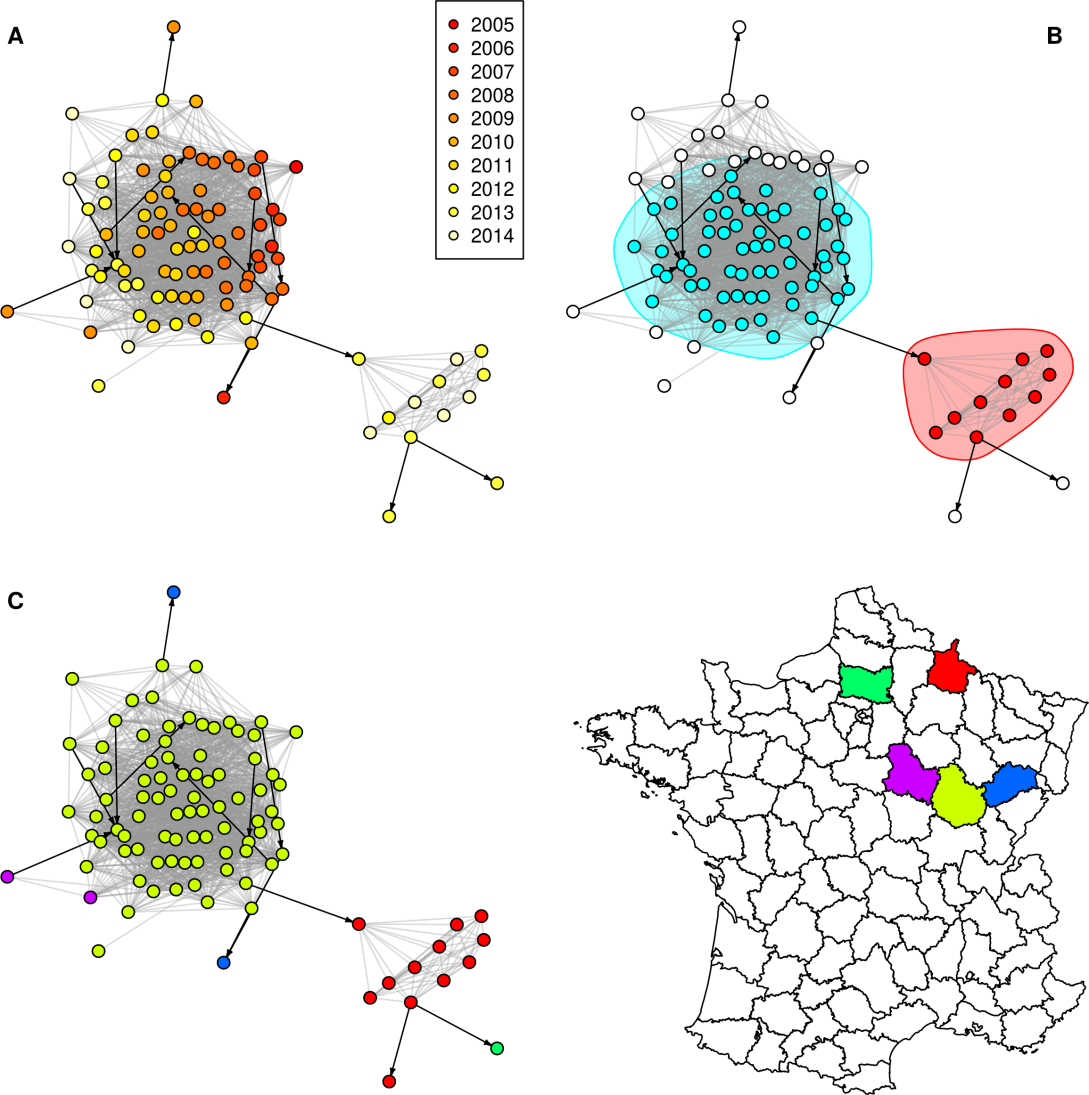
Component 1 of the SB0120 network. Grey: spatial neighbourhood links; black: network neighbourhood links; nodes are coloured according to (A) the bTB notification year; (B) the community (only communities of more than 10 nodes within the component are taken into account); (C) the department. Node locations are identical in A, B and C.

**Fig 2:**
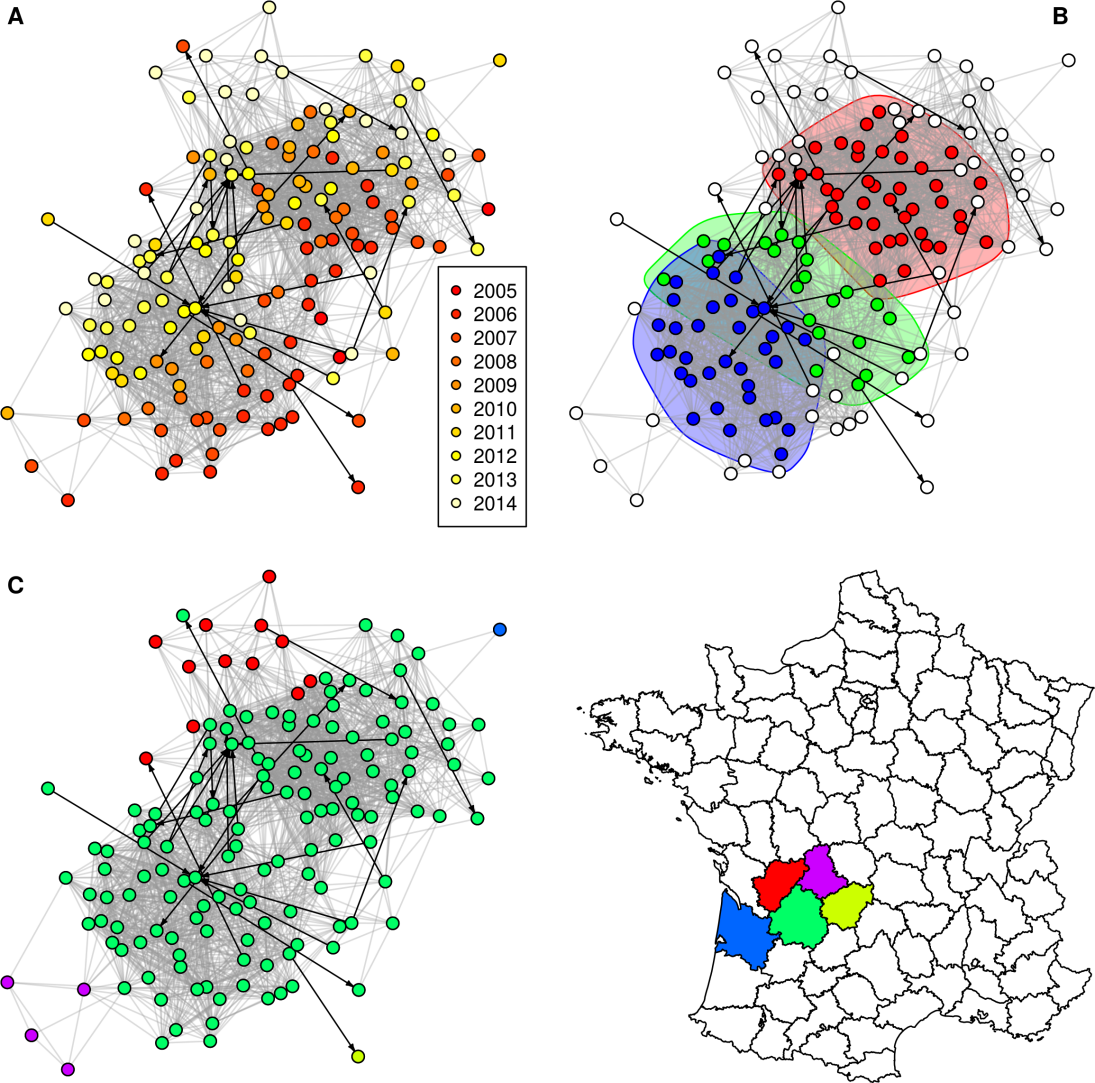
Component 2 of the SB0120 network. Grey: spatial neighbourhood links; black: network neighbourhood links; nodes are coloured according to (A) the bTB notification year; (B) the community (only communities of more than 10 nodes within the component are taken into account); (C) the department. Node locations are identical in A, B and C.

**Fig 3:**
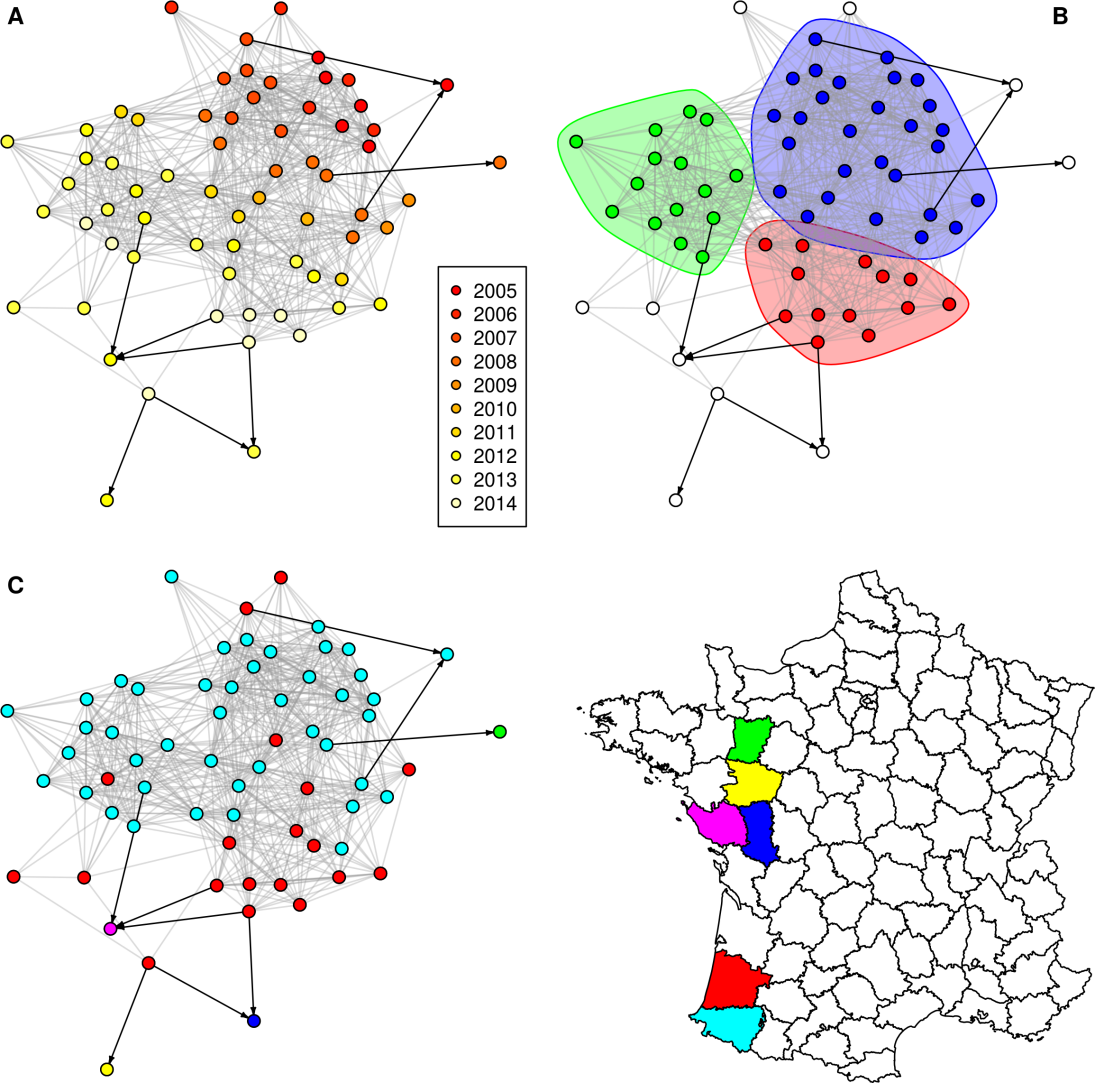
Largest component of the SB021 network. Grey: spatial neighbourhood links; black: network neighbourhood links; nodes are coloured according to (A) the bTB notification year; (B) the community (only communities of more than 10 nodes within the component are taken into account); (C) the department. Node locations are identical in A, B and C.

The **Fig 1** shows the component 1 of the SB0120 network (see **Fig 2** and **Fig 3** and **S7 Fig** and **S8 Fig** for the others networks) with nodes coloured according to the year of bTB notification (top left), according to the community (top right–only communities of more than 10 nodes are coloured) or according to the department (bottom). As expected (because of the time constraint of five years taken into account when defining the links between nodes), infected herds appear grouped by detection year in Figs 1–3, in particular for the component 2 of the SB021 network (**Fig 3**).

In both large components of the SB0120 network and in the unique large component of the SB021 network, the edge betweenness was markedly and significantly higher in the network neighbourhood links than in spatial neighbourhood links (**Table 5**). In Figs 1–3, edges representing network neighbourhood are represented in black, directed arcs, whereas edges representing spatial neighbourhood are represented in grey, undirected arcs (for simplicity, as these edges are bidirectional). This representation allows illustrating the difference of edge betweenness between edge types: some network neighbourhood edges clearly connect separate communities such as in the component 1 of the SB0120 network (**Fig 1**).

**Table 5:** Average value of edge betweenness in network neighbourhood links and in spatial neighbourhood links, for each component of more than 10 nodes within each network.

		Average edge betweenness of network neighbourhood links	Average edge betweenness of spatial neighbourhood links	*p*-value (Wilcoxon’s test)
SB0120				
	**Component 1**	**123.8**	**3.6**	**< 0.001**
	**Component 2**	**129.8**	**10.7**	**< 0.001**
SB021				
	**Component 1**	**32.2**	**5.1**	**< 0.001**
SB0134				
	Component 1	17.4	5.5	0.19
	Component 2	-	2.5	-

Finally, the proportion of links connecting distinct communities was calculated for both types of links. For the two large components of the SB0120 network, the proportion of network neighbourhood links connecting distinct communities (85% of network neighbourhood links for component 1 and 100% for component 2) was significantly higher than for spatial neighbourhood links (24% of spatial neighbourhood links for component 1 and 48% for component 2) (Fisher’s test, p<0.001). The same significant difference was observed for the largest component of the SB021 network with 78% of the network neighbourhood links connecting distinct communities, against 31% for the spatial neighbourhood links which were between-cluster links (p = 0.006). No such difference was observed for the component 1 of the SB0134 network (p = 0.164). As the component 2 of the SB0134 network did not include any network neighbourhood links (**Table 4**), this difference could not be assessed.

## 4. Discussion

The aim of this study was to analyse the role of cattle movements in bTB spread in France between 2005 and 2014, using social network analysis and logistic regression models. First, at a national scale, we showed a statistical association between a higher in-degree (OR = 2.4 [1.1–5.4]) or a higher ingoing contact chain (OR = 2.2 [1.0–4.7]) and the bTB infection status of cattle herds. Indeed, both indicators are correlated and a higher in-degree (or a higher ingoing contact chain) induces an increased probability to purchase an infected animal [9].

Secondly, at a more local scale, we compared the role of cattle movements with the role of the spatial neighbourhood. Exposure to bTB-infected herds by cattle movements had a weaker impact on the herd bTB status than exposure to bTB-infected herds from the spatial neighbourhood. Only purchases of animals directly from infected herds (neighbourhood of order 0 in the network neighbourhood) were shown to be associated with bTB infection (OR = 2.9 [1.7–5.2]). On the contrary, an association between bTB infection and the presence of bTB-infected herds in the spatial neighbourhood was observed up to 3 communes away (neighbourhood of orders 0 to 3 in the spatial neighbourhood). The ORs decreased from 4.2 [2.8–6.5] for the exposure to a bTB-infected herd located in the same commune as the studied herd, to 1.7 [1.1–2.6] for the exposure to a bTB-infected herd located 3 communes away. Since the mean area of a French commune is about 16 km^2^ (equivalent to a square of 4km x 4km), this would mean an increased risk of bTB infection up to roughly 12km around an infected herd. It is consistent with the hypothesis of local transmission of bTB strains (distances < 14 km) in Spain put forward by de la Cruz *et al*. [28]. However, it is somewhat higher (but of the same order of magnitude) than the 6km radius of high-risk area around infected herds described in Great Britain by Green *et al*. [29], which could be explained by differences in the level of farm fragmentation and use of pasture between Great Britain and France [30]. We also estimated population attributable fractions associated with the network neighbourhood (i.e. cattle movements) and the spatial neighbourhood. The population attributable fraction for cattle movements was 12% [5%–18%], while the one for spatial neighbourhood was much higher (73% [68%–78%]). These population attributable fractions were consistent with the 16% (respectively, 75%) of herd infections attributed to cattle movements (respectively, local effects) in Great Britain in 2004 by Green *et al*. [29]. However, as showed by Brooks-Pollock *et al*. in 2014 [31] for Great Britain, it is possible that most of the herd breakdowns have infections due to multiple causes (cattle movements and local transmission–as whole-herd culling is the default control measure in France, recurrence due to missed infected animals is unlikely). Furthermore, local transmission may include direct contacts despite the fences, contacts with infected wildlife or unrecorded local movements between herds [32].

Finally, we looked at networks of potential effective contacts between bTB-infected herds, for the three major spoligotypes reported in France (SB0120, SB021 and SB0134). Cattle movements (i.e. network neighbourhood edges) were often links connecting distinct communities and sometimes distinct geographical areas. This is consistent with the hypothesis that cattle movements would allow the disease to spread into bTB-free areas, whereas local transmission (e.g. contacts on pastures or contacts with infected wildlife) would be involved in the within-area spread. Therefore, although cattle movements seemed to quantitatively have a weaker role than spatial neighbourhood, they were essential to the overall bTB dynamics in France.

The role attributed to cattle movements in bTB spread varies according to the country (or area) and the infection level. For example, in Scotland, a low-incidence area, Gates *et al*. [33] showed that the most likely source of bTB infection was cattle imported from endemic areas. In England, Carrique-Mas *et al*. [6] concluded that cattle movements were the only bTB source for restocked herds with no bTB history; and Gilbert *et al*. [34] showed in 2005 that the variables linked to cattle movements were the best predictors of the infection state. Ribeiro-Lima *et al*. [9] explained that the main cause of the infection of 12 cattle herds in Minnesota (the United States) by the end of 2008 was also cattle movements. On the other hand, in Ireland, Clegg *et al*. [35] estimated that only 6% of herd restrictions could be attributed to a recent introduction of an infected animal. In Michigan (the United States), Okafor *et al*. [36] established that cattle movements were not the most likely source of infection for most of the infected herds. This suggests a difference in the mechanisms involved in bTB spread function of the geographical area and a stronger role of cattle movements in low-incidence areas than in high-incidence ones [29,32,33,35]. The difference can also be related to the strains of the different countries, as Humblet *et al*. [37] observed a higher risk linked to cattle movements only for the predominating strain type SB0162 in Belgium. Since these two hypotheses are not mutually exclusive, the difference can also be due to a mixed of both: different mechanisms between low- and high-incidence areas and different spoligotypes with an evolution over time for the incidences and the spoligotypes.

A 5-year period of time was taken into account to compute the network indicators. Indeed, bTB transmission is low and detection of the infection difficult: an infected animal can have been infectious for several years before being detected. This obliged us to consider a long period of time to compute networks indicators. On the other hand, other disease outbreaks occurred during this period in France, like the Bluetongue epidemic in 2007–2008. This could have bias our results. However, Dutta *et al*. [7] showed that there was no significant difference in the distributions of network indicators between the networks aggregated yearly from 2005 to 2009. Hence, it is unlikely that the Bluetongue outbreak in 2007–2008 impacted our results.

In France, when a herd is reported as bTB-infected, epidemiological investigations are implemented in order to investigate the bTB status of all the herds it has been in contact with, e.g. herds which purchased cattle from the infected one, herds which sold cattle to the infected one, or herds located or having pastures close to the infected one. Thus, it is more likely to report bTB infection in a herd that had a direct contact with an infected herd than in an isolated herd. This could therefore impact our results, artificially increasing the number of herds reported as bTB-infected herds in the neighbourhood of case herds (and not of control herds). However, as herds from both the network neighbourhood and spatial neighbourhood are investigated during these epidemiological investigations, it is unlikely that this biased the relative importance of cattle movements and local transmission estimated in our study.

The spoligotype was the only molecular marker for which typing results were available for almost all herds reported as infected between 2005 and 2014. However, this marker is considered very stable, and has sometimes a limited discriminative power (especially when a dominant strain exists) [38]. This may lead to an overestimation of the role of cattle movements or spatial neighbourhood: indeed, we assumed a relation between infected herds as they shared the same spoligotype, whereas this relation may not have existed in the reality. Nevertheless, taking into account different spoligotypes as different outbreaks was one of the strengths of this study. In the future, these analyses could be improved using molecular markers having a higher discriminative power, like VNTR or MLST [39,40].

Based on the results of this study, it seems that control measures implemented in case of cattle movements could limit the spread of the disease from bTB-infected areas to bTB-free areas. As an example, Defra recently proposed to introduce in England statutory post-movement bTB testing of cattle moved from a bTB high-risk area to a bTB low-risk area [41]. However, skin tests have a limited sensitivity. A recent meta-analysis of the VLA [42] estimated the median sensitivity of the single intradermal skin test at 0.94, but with a large 95% confidence interval between 0.49 and 1.00. Since pre- or post-movement tests are individual tests, they may lead to many false negative results, in particular after physiological stress. Thus, control measures based on cattle movements alone may produce limited results.

On the other side, as local transmission seems to play a strong role in the spread of bTB, control measures have also to focus on this type of transmission. Local transmission consists of at least three different effective contacts: contacts with other infected animals at pasture despite the fences, contacts with infected wildlife or environment and unreported local movements. In France, it is unknown which component of local transmission is the most important and which control measures are the most effective. Hence, more studies about each of local transmission, especially on neighbouring effective contacts at pasture, are needed. Nevertheless, as showed by Brooks-Pollock *et al*. [31] for Great Britain, control measures may focus on several transmission routes to effectively control bTB spread.

## 5. Conclusion

The aim of this study was to assess the role of cattle movements in the spread of bTB in France between 2005 and 2014. Three analyses were performed. At a national scale, we showed a statistical association between several centrality measures in the cattle trade network and the bTB infection status of cattle herds. At a more local scale, we estimated the relative importance of cattle movements and local transmission in bTB spread and showed that cattle movements had a lower role compared to spatial neighbourhood (i.e. local transmission): the population attributable fraction associated with the exposure to bTB infection in the network neighbourhood was of 12% *versus* 73% for the one associated with the exposure to bTB infection in the spatial neighbourhood. However, when analysing, for several spoligotypes, the networks of potential effective contacts between bTB-infected herds, we observed that trade links were frequently the way to connect distinct communities of bTB-infected herds. Therefore, although their role was quantitatively lower than that of spatial neighbourhood, cattle movements appeared to be essential in the French bTB dynamics between 2005 and 2014.

## Supporting Information

S1 Fig Distribution of the coefficients of the multivariate logistic regression model including the in-degree; 1,000 permutations (bootstrap). Red dotted line: coefficient for the real network; black dotted lines: the 5^th^ and the 95^th^ percentiles.
(TIFF)

S2 Fig Distribution of the coefficients of the multivariate logistic regression model including the ingoing contact chain; 1,000 permutations (bootstrap). Red dotted line: coefficient for the real network; black dotted lines: the 5^th^ and the 95^th^ percentiles.
(TIFF)

S3 Fig The ROC curve for the multivariate logistic regression model of bTB infection status according to network centrality indicators: complete model including the in-degree. (TIFF)

S4 Fig The ROC curve for the multivariate logistic regression model of bTB infection status according to network centrality indicators: complete model including the ingoing contact chain. (TIFF)

S5 Fig The ROC curve for the multivariate logistic regression model of bTB infection status according to the exposure to bTB-infected herds in the network and spatial neighbourhood (TIFF)

S6 Fig Spatial distribution of the mean deviance residuals per commune for the multivariate logistic regression model of bTB infection status according to the exposure to bTB-infected herds in the network and spatial neighbourhood. commune: the smallest administrative French subdivision.
(TIFF)

S7 Fig Component 1 of the SB0134 network. Grey: spatial neighbourhood links; black: network neighbourhood links; nodes are coloured according to (A) the bTB notification year; (B) communities of more than 10 nodes within the component; (C) the department. Node locations are identical in A, B and C.
(TIFF)

S8 Fig Component 2 of the SB0134 network with more than 10 nodes. Grey: spatial neighbourhood links; black: network neighbourhood links; nodes are coloured according to (A) the bTB notification year; (B) the community (only communities of more than 10 nodes within the component are taken into account); (C) the department. Node locations are identical in A, B and C.
(TIFF)

S1 File Definitions of herd types. (DOCX)
