# Supplementary figures and images for: Role of Cattle Movements in Bovine Tuberculosis Spread in France between 2005 and 2014
Source: PLoS One. 2016 Mar 28;11(3):e0152578. doi: 10.1371/journal.pone.0152578 (PMC4809620; doi:10.1371/journal.pone.0152578)

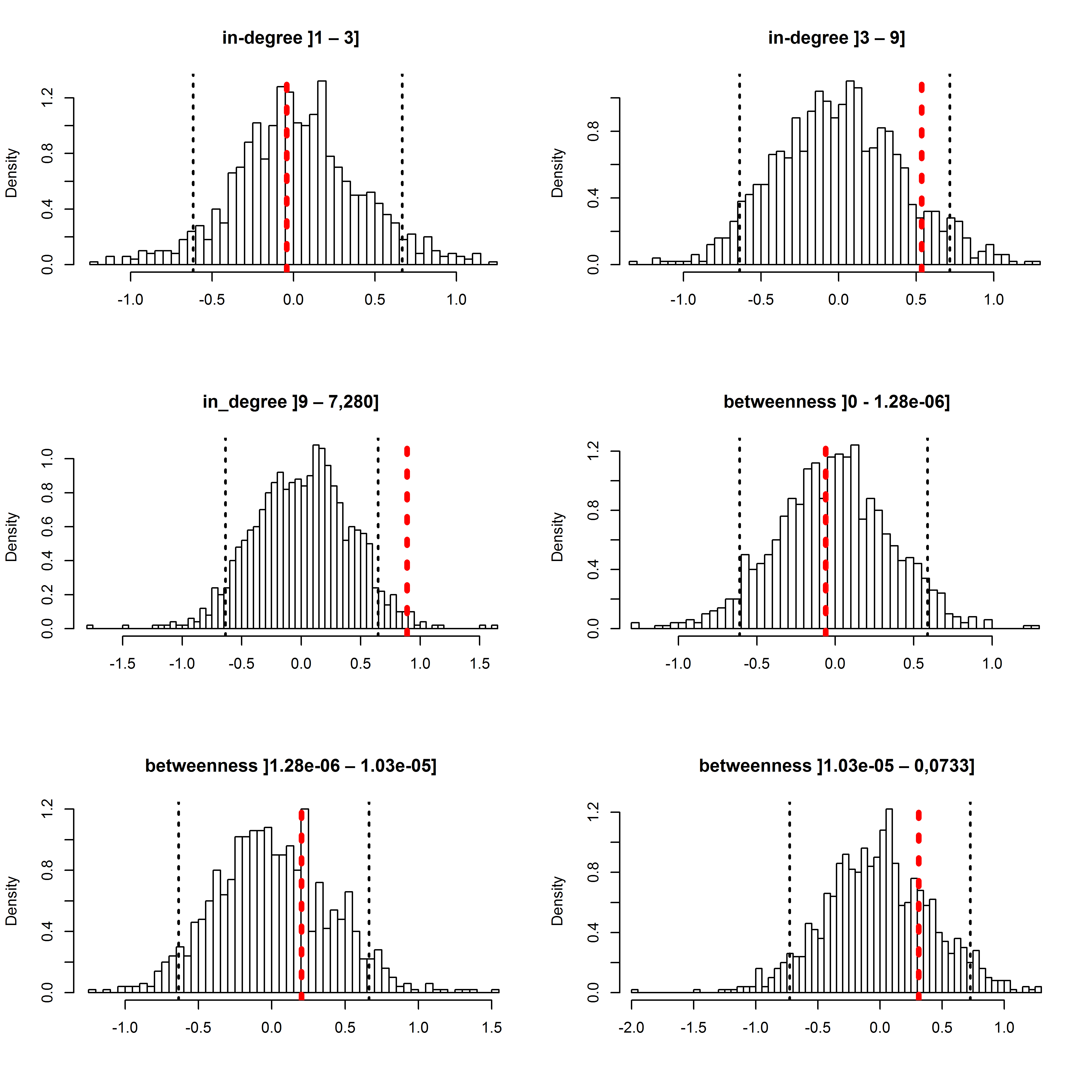

Supplement: S1 Fig — Red dotted line: coefficient for the real network; black dotted lines: the 5th and the 95th percentiles. (TIFF) [file pone.0152578.s001.tiff]

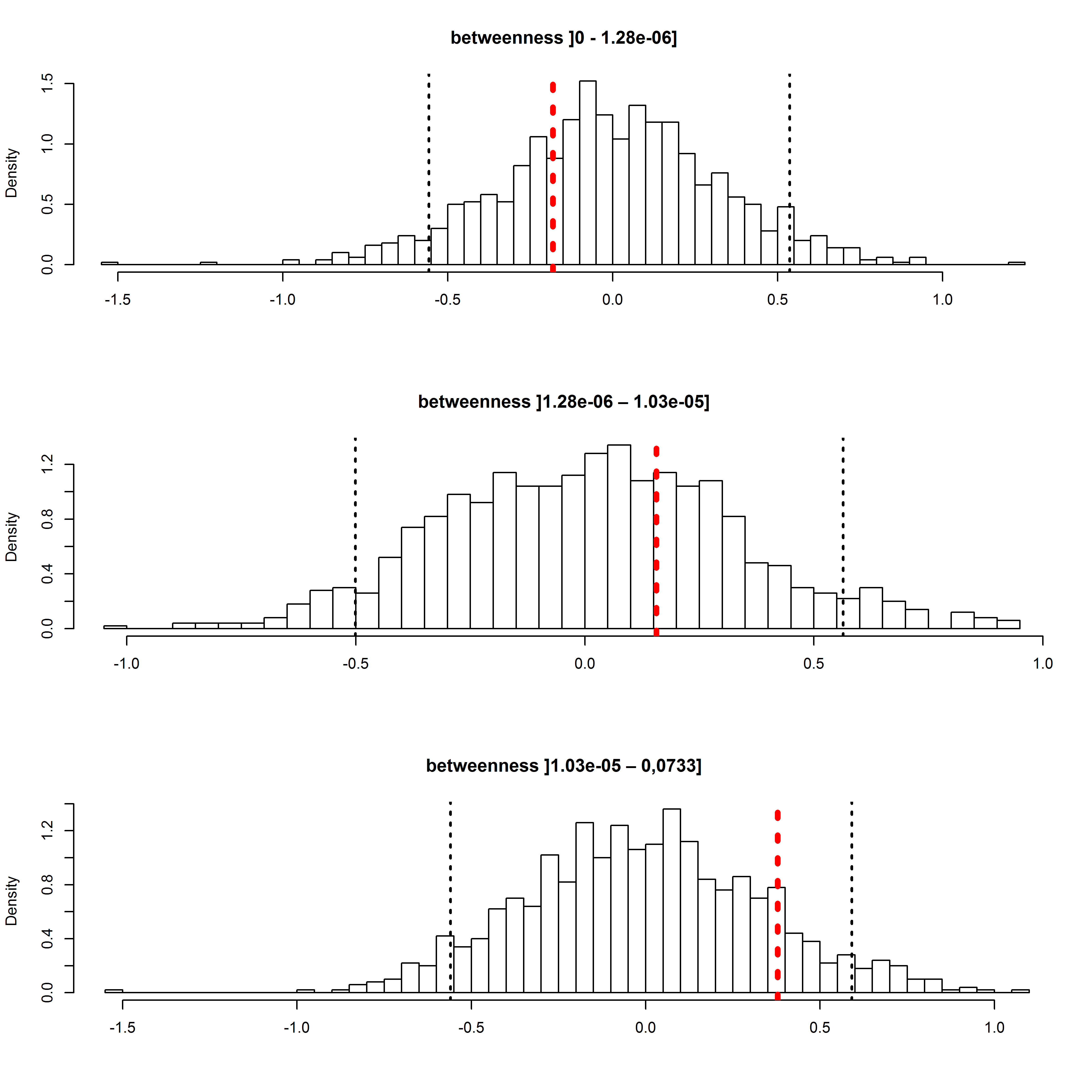

Supplement: S2 Fig — Red dotted line: coefficient for the real network; black dotted lines: the 5th and the 95th percentiles. (TIFF) [file pone.0152578.s002.tiff]

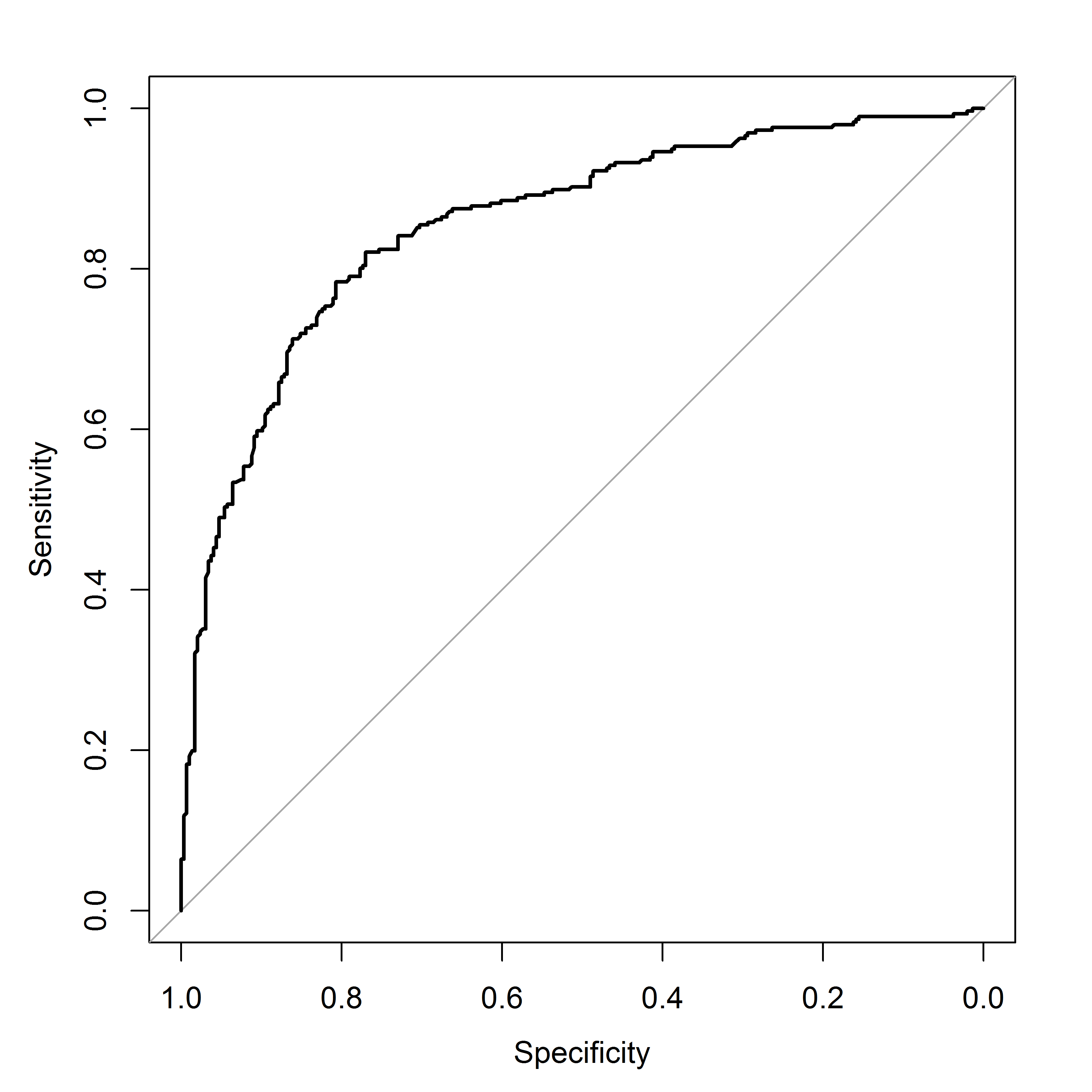

Supplement: S3 Fig — (TIFF) [file pone.0152578.s003.tiff]

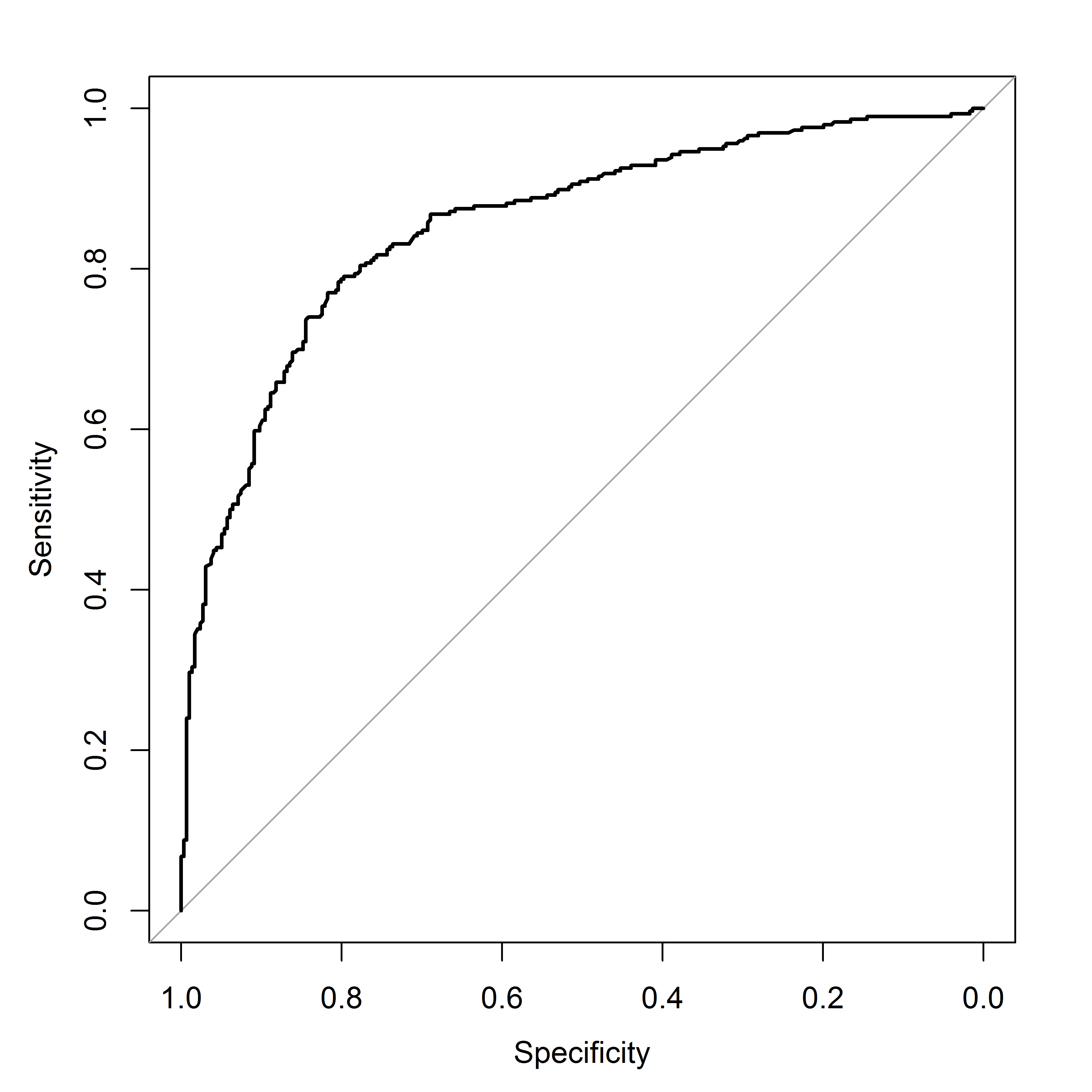

Supplement: S4 Fig — (TIFF) [file pone.0152578.s004.tiff]

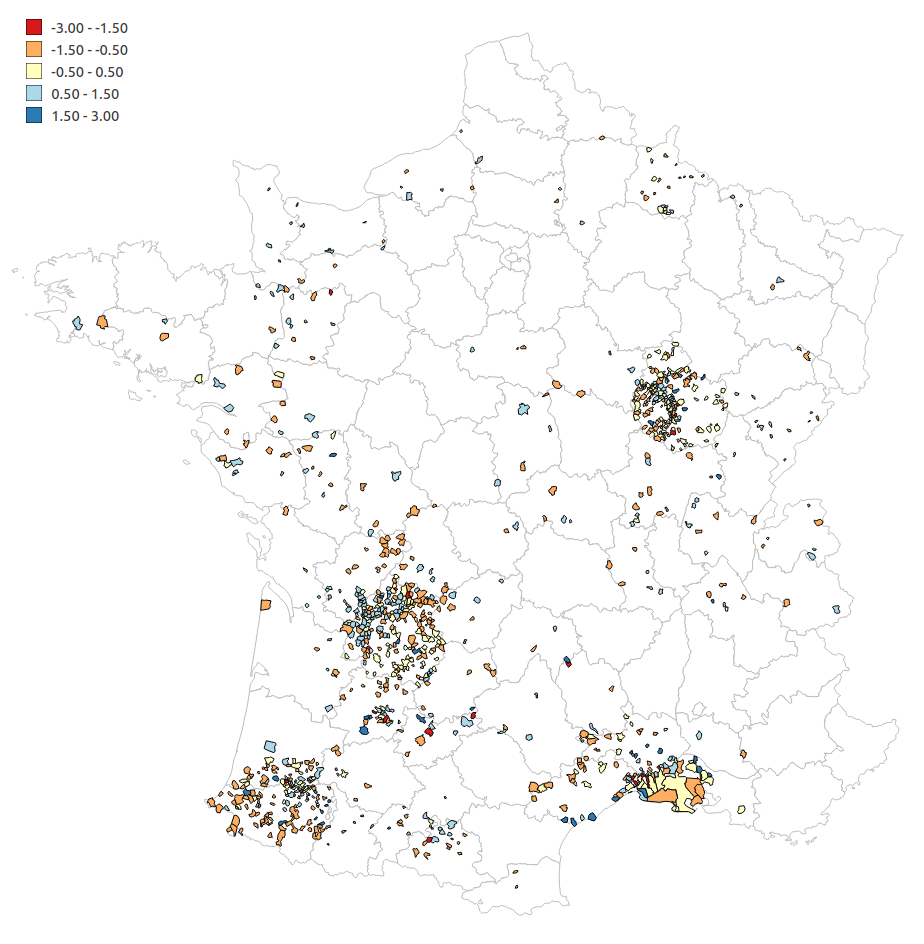

Supplement: S6 Fig — commune: the smallest administrative French subdivision. (TIFF) [file pone.0152578.s006.tiff]

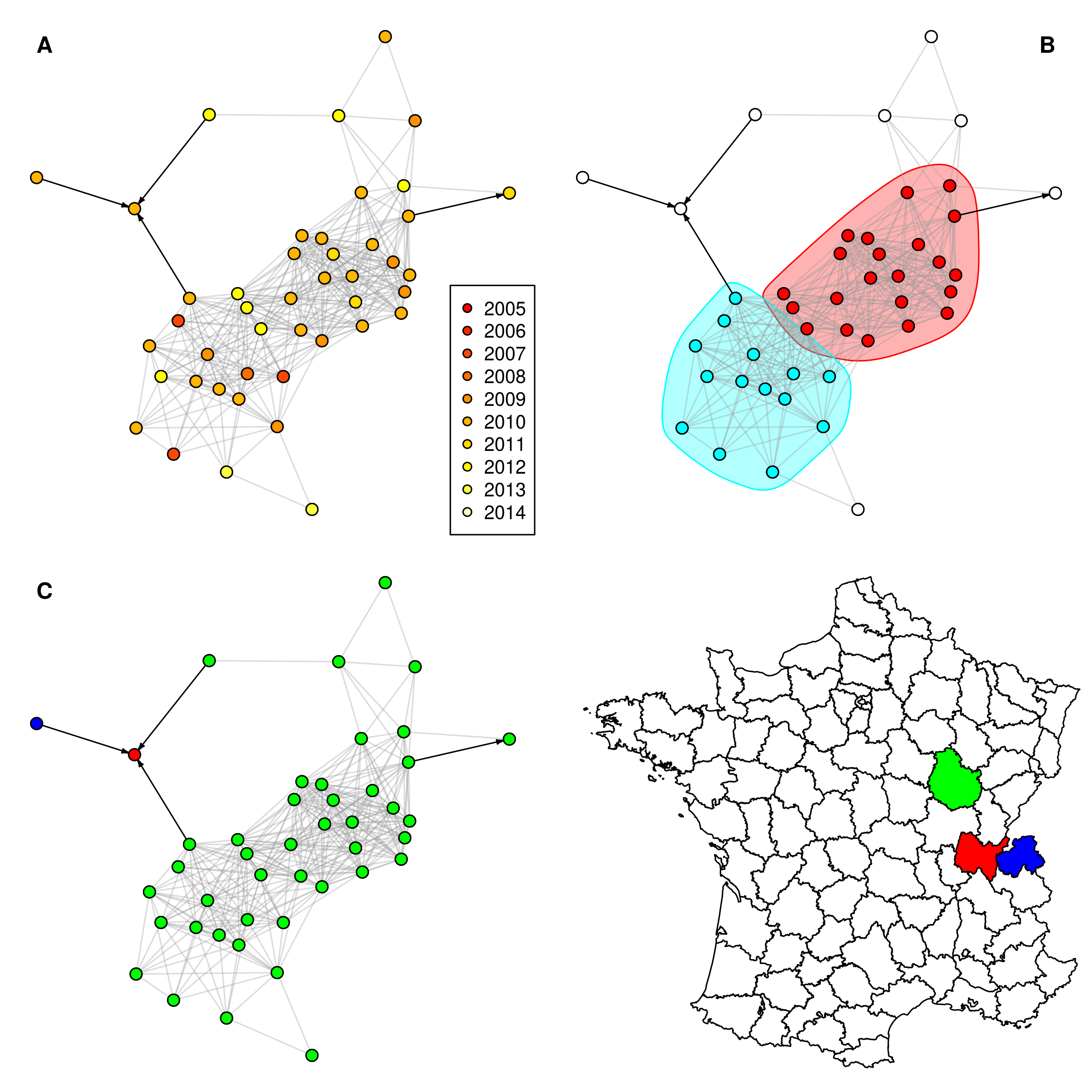

Supplement: S7 Fig — Grey: spatial neighbourhood links; black: network neighbourhood links; nodes are coloured according to (A) the bTB notification year; (B) communities of more than 10 nodes within the component; (C) the department. Node locations are identical in A, B and C. (TIFF) [file pone.0152578.s007.tiff]

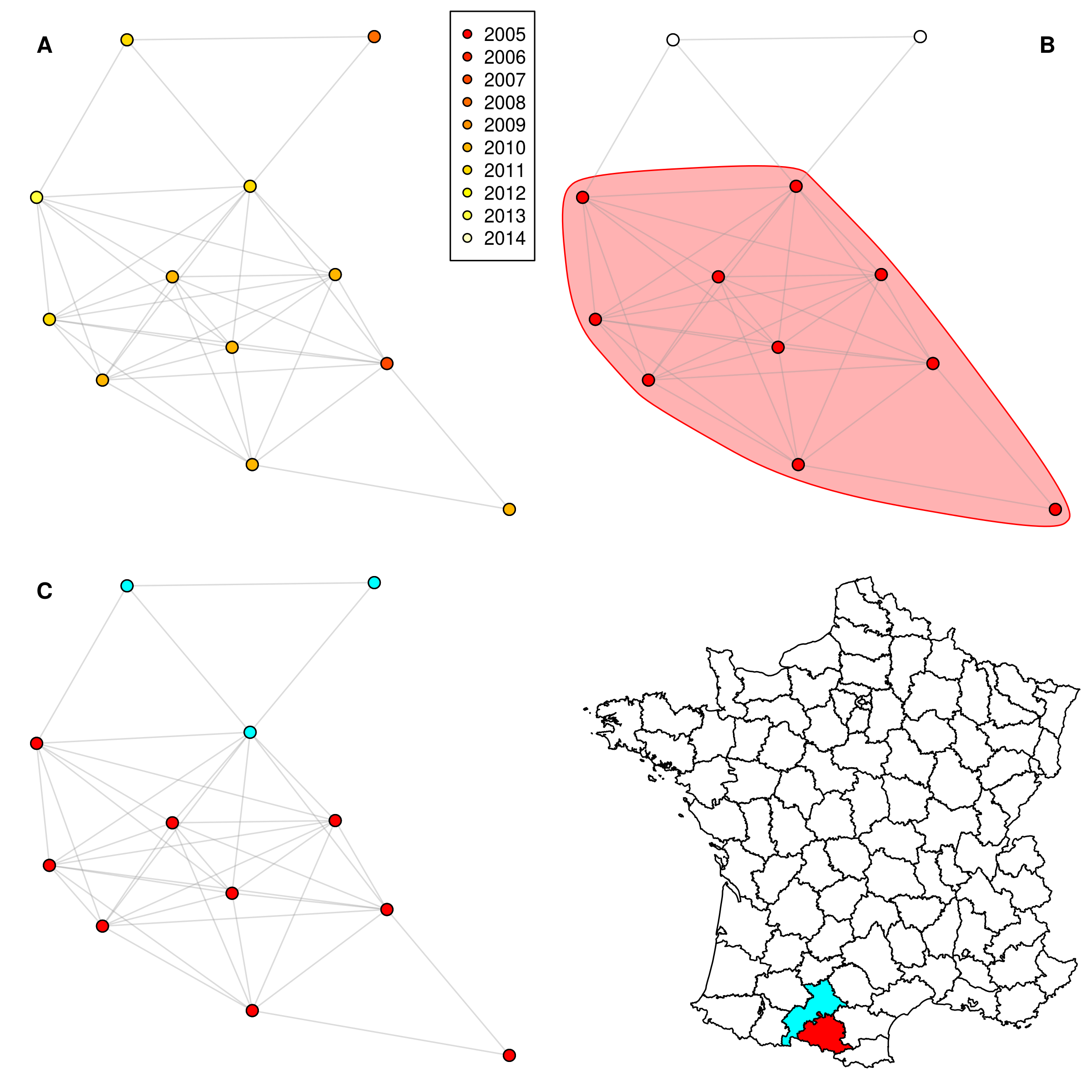

Supplement: S8 Fig — Grey: spatial neighbourhood links; black: network neighbourhood links; nodes are coloured according to (A) the bTB notification year; (B) the community (only communities of more than 10 nodes within the component are taken into account); (C) the department. Node locations are identical in A, B and C. (TIFF) [file pone.0152578.s008.tiff]
