# Supplementary material for: Role of Cattle Movements in Bovine Tuberculosis Spread in France between 2005 and 2014
Source: PLoS One. 2016 Mar 28;11(3):e0152578. doi: 10.1371/journal.pone.0152578 (PMC4809620; doi:10.1371/journal.pone.0152578)
Supplement: S1 File — (DOCX) [file pone.0152578.s009.docx]

**S1 File. Definitions of herd types.**

The definitions of herd types are:

- Small herd: herd with less than 10 calvings, less than 10 females over two years of age and less than 10 males sold to slaughterhouse per production campaign,

- Fattening herd: herd with less than 10 calvings and more than 10 males sold to slaughterhouse per production campaign,

- Dairy herd: herd with more than 10 calvings of dairy and mixed females and less than 10 calving of beef females per production campaign,

- Mixed herd: herd with more than 10 calvings of dairy females and more than 10 calvings of beef females per production campaign,

- Beef herd: herd with more than 10 calvings of beef and mixed females and less than 10 calvings of dairy females per production campaign,

- Other herd: herd which does not meet the above criteria.
